# Revision of the world species of *Zambion* (Hymenoptera, Ichneumonidae, Tryphoninae)

**DOI:** 10.3897/zookeys.159.2219

**Published:** 2011-12-23

**Authors:** Andrew M.R. Bennett, Diana I. Barnes

**Affiliations:** 1 Agriculture and Agri-Food Canada, Canadian National Collection of Insects, 960 Carling Avenue, Ottawa, Ontario, Canada K1A 0C6

**Keywords:** *Zambion*, Ichneumonidae, Tryphoninae, Afrotropical, revision

## Abstract

The world species of *Zambion* Kasparyan (Hymenoptera: Ichneumonidae: Tryphoninae) are revised including re-descriptions of *Zambion monodon* Kasparyan and *Zambion hirtum* Delobel. Five new species are described: *Zambion kasparyani* **sp. n.**, *Zambion rogeri* **sp. n.**, *Zambion eileenae* **sp. n.**, *Zambion wahli* **sp. n.** and*Zambion broadi* **sp. n.** A key to species is provided. The genus is endemic to Africa (Angola to Kenya) and is one of only three genera of the tribe Tryphonini recorded from the Afrotropical region.

## Introduction

The tribe Tryphonini is poorly represented in the Afrotropical region with only 3 of 21 extant genera recorded (Yu et al. 2009; Bennett 2002): *Boethus* Förster (Scaramozzino 1991) and two endemic genera: *Ibornia* Seyrig (Seyrig 1935) and *Zambion* Kasparyan (Kasparyan 1993). In total, there are only eight described species of Tryphonini recorded from the Afrotropical region: two species each of *Boethus* and *Zambion* and four species of *Ibornia* (Yu et al. 2009). It was speculated by Gauld (1987) that a reason for the lack of ctenopelmatine ichneumonids from tropical regions is because their hosts (sawflies) are rare in the tropics in terms of species and biomass. The same hypothesis could explain the low number of species of Afrotropical Tryphonini because almost all known host records for the tribe are from sawflies (Gauld et al. 1997). The hosts of *Ibornia* and *Zambion* are not known, but they likely parasitize tenthredinid sawflies based on the known host range of related genera (e.g., *Dyspetes* Förster and *Otoblastus* Förster) (Gupta 1983; Kasparyan 1973). Of the 10 genera of Tryphonini for which hosts are known, only two have been reared from hosts other than Tenthredinidae: *Boethus* species have been consistently reared from Argidae (Hymenoptera) (Gauld et al. 1997) and some species of *Grypocentrus* Ruthe have been reared from Eriocraniidae (Lepidoptera) (Jordan 1998) (most rearing records of *Grypocentrus* are from Tenthredinidae) (Kasparyan 1973).

Apart from the presumed lower number of potential hosts, another factor that definitely contributes to the small number of species of Afrotropical Tryphonini (and Ichneumonidae in general) is the relatively little amount of taxonomic research on the ichneumonids of the region. In other regions such as the Neotropical, recent active research programmes have greatly elucidated the ichneumonid fauna (e.g., Gauld 1991; Gauld et al. 1997, 2000, 2002). In the Afrotropical region, some excellent work has been done in the past, for example, Seyrig (1932, 1934, 1935, 1952) on various subfamilies, Heinrich (1967a, b, c, 1968a, b) on Ichneumoninae, and Gauld and Mitchell (1978) on Ophioninae, but recently, taxonomic studies including Afrotropical species have been uncommon. For example, Yu et al. (2009) listed only 8 taxonomic papers that included Afrotropical species published between 2000 and 2008 (compared to 65 for the Neotropical region and 46 for the Oriental). In terms of described species, the Afrotropical ichneumonid fauna is not as speciose as the Neotropical and Oriental regions (1,946 described species in the Afrotropical compared to 3,803 in the Neotropical and 3,595 in the Oriental) (Yu et al. 2009). But based on examination of unidentified material at the American Entomological Institute, Canadian National Collection of Insects and the Natural History Museum, London, there are large numbers of undescribed species in the Afrotropical region, especially from the tropics and South Africa. Without additional taxonomic studies of the fauna, well-founded assessments of species diversity and biological patterns for sub-Saharan Africa will be difficult to achieve. The purpose of this paper is to revise the genus *Zambion* including description of five new species. It is hoped that the revision of such a beautiful and morphologically distinct genus will encourage other researchers to spend much-needed time working on this interesting, but under-studied fauna.

## Methods

All terms of ichneumonid morphology follow Townes (1969) with the following modifications: hypostomal carina for ‘oral carina’, supra-antennal area for ‘frons’, supraclypeal area for ‘face’, gena for ‘temple’, occiput for ‘postocciput’, malar space for ‘cheek’, epicnemial carina for ‘prepectal carina’, laterotergites for ‘epipleura’, gonoforceps for ‘claspers’, and hypopygium for ‘subgenital plate’. The term ‘mesosoma’ is used for the body region that includes the thorax and first abdominal segment (the propodeum). The term ‘metasoma’ is used for the apparent abdomen, with MS1, MS2, etc. referring to metasomal segments 1, 2, etc., T1, T2, etc. referring to the tergites of metasomal segments 1, 2, etc. and S1 referring to the sternite of metasomal segment 1. The term T2+ refers to tergite 2 and all tergites posterior to T2. Terms of relative position of the body follow Goulet and Huber (1993). Wing venation terms follow the Comstock-Needham system as updated by Ross (1936) and incorporate the recommendations of Goulet and Huber (1993) except for naming of the vein that forms the distal edge of fore wing cell 1+2Rs (the ‘areolet’ of Townes 1969). This vein is of uncertain origin and is here referred to as ‘vein 3rs-m’ in conformity with Wahl and Gauld (1998). The following terms for specialized structures are defined: epomia: a raised ridge (carina) on pronotum (Figs 30–31); glymma: lateral depression sub-basally on T1; notaulus: longitudinal groove sublaterally on mesoscutum (Fig. 29); sternaulus: longitudinal groove subventrally on mesopleuron; ventral transverse carina of propodeum: a carina that extends from the metasomal foramen of the propodeum to the pleural carina (Fig. 35).

Measurements for particular structures were made as follows: supraclypeal width was measured at midheight compared to height in the middle (laterally); horn length measured in dorsal view versus pedicel width at middle in lateral view; gena width measured at lower (narrowest) point versus transverse width of eye at midheight (both in lateral view); length of scutellar carina measured from base of scuto-scutellar groove to apex of scutellum; point of union of posterior transverse carina of propodeum (ptc) and pleural carina (pc) was estimated by measuring the distances shown in Figs 35–37: from the propodeal spiracle to the ptc and from the ptc to the ventral transverse carina (vtc) (or in *Zambion monodon* and *Zambion kasparyani*, where the vtc would be, if present); hind femur dimensions were calculated by dividing the maximum length including the distal trochantellus by the height at midpoint; length of T1 measured in lateral view compared to width of T1 measured in dorsal view at posterior edge. Measurements of holotypes are shown in parentheses after range. Characters listed in the generic description are those deemed relevant to distinguish *Zambion* from all other genera of Tryphonini as defined by Kasparyan (1973), Kasparyan and Tolkanitz (1999) and Bennett (2002).

Specimens are deposited at the following institutions (acronyms according to Evenhuis 2011):

Canadian National Collection of Insects (CNC); The Natural History Museum, London (NHM); Muséum national d’Histoire naturelle, Paris (MNHN), Finnish Museum of Natural History, Helsinki (MZH), California Academy of Sciences, San Francisco (CAS). Label data for holotypes is reported *verbatim* from the labels, with our comments about the labels indicated in square brackets. Locality data for other specimens are provided in a standardized format.

Digital photos were taken using a Leica MZ16 stereomicroscope with motorized focus drive attached to a Leica DFC420 digital camera. Photos were combined and edited using Leica Application Suites Montage Multifocus software V3.8, Auto-Montage Pro 5.01 and Adobe Photoshop CS4. Authorship of all species is by Bennett and Barnes.

## Taxonony

### Zambion

Kasparyan

http://species-id.net/wiki/Zambion

Zambion  Kasparyan, 1993: 86. Type: *Zambion monodon* Kasparyan. By monotypy.

#### Diagnosis.

Differentiated from other genera of Tryphoninae by combination of both of the following characters: 1) unidentate mandibles (Fig. 22); 2) supra-antennal area with a medial horn (not a vertical lamella) slightly ventral to medial ocellus (Figs 16–21, 24–27). *Zambion* would most easily be mistaken for *Ibornia* Seyrig, but *Ibornia* has a supra-antennal vertical lamella instead of a horn and T1 and T2 are fused (not fused in *Zambion*).

#### Description.

**Adult.** Fore wing length 4.9 to 7.2 mm. Clypeus separated from supraclypeal area by a strong groove (most species) (Fig. 11) or weakly separated in *Zambion hirtum* Delobel (Fig. 10); strongly convex or moderately flat in profile; not divided into dorsal and ventral faces by a transverse line (most species) (Fig. 11) or divided (Fig. 12). Apical edge of clypeus not projecting ventroanteriorly in lateral view, truncate to slightly convex medially in anterior view, without a pair of medial denticles. Mandible unidentate (Fig. 22), outer face (anterior surface) basally flat and coarsely punctate (not inflated and impunctate as in *Ctenochira*). Glossa slightly to strongly elongated (Fig. 15). Posterior mandibular condyles separated by greater width than inner margins of eyes at level of clypeal foveae. Malar space 0.4 to 0.7 times basal width of mandible. Occipital carina present only dorsally (between inner margins of eyes), therefore not joining hypostomal carina, the latter not directed posteriorly into a tooth. Supra-antennal area with or without a glabrous depression, if present then just as a deep dimple directly ventral to medial ocellus, not an elongate groove as in *Cosmoconus* Förster. Supra-antennal area with a medial horn (Figs 16–21, 24–27). Vertex posterior to medial ocellus without a rounded, conical protuberance. Distance between inner edges of toruli 0.1 to 0.4 times width of one torulus.

Dorsal edge of pronotum (seen in dorsal view) strongly thickened laterally (Fig. 28). Epomia present only as a slight sharpening of ventroanterior ridge of pronotum (not crossing pronotal groove) (Figs 30–31). Notaulus weak (Fig. 29) or absent. Subtegular ridge slightly protruding laterally, not extending dorsally as a vertical lamina that nearly touches tegula, without a posterior longitudinal slot. Epicnemial carina not joined by an auxiliary carina that extends from anterior edge of mesopleuron. Vertical portion of epicnemial carina close to anterior edge of mesopleuron and dorsally curving anteriorly to meet anterior edge of mesopleuron (Figs 32–33). Longitudinal carinae of mesopleuron and mesoscutum (as in *Ibornia*) absent. Sternaulus weak and wide to 0.2 to 0.3 length of mesopleuron. Angular flange on posterolateral edges of mesoscutum and associated ‘axillary tongue’ both present and strongly angulate. Scutellum completely black (most species), completely yellow (*Zambion hirtum* and *Zambion kasparyani*) or black with yellow apically (*Zambion monodon*), lateral carinae present only basally or up to 0.5. Propodeum with all carinae present except anterior transverse carina completely absent in all species (Fig. 34), medial longitudinal carinae absent anteriorly in *Zambion wahli* and *Zambion broadi* and posterior transverse carina interrupted medially in the males of *Zambion wahli* and *Zambion broadi*. Posterior transverse carina angulate where it intercepts medial longitudinal carinae (Fig. 34). Foretibia with a strong tooth on apex of anterior side or tooth weak to absent. Trochantelli not fused on middle and hind legs. Hind tarsal claws stoutly pectinate in basal 0.4 to 0.6. Fore wing vein 3rs-m present. Cell 1 + 2Rs subrectangular or subrhombic, width not greater than 1.4 times height, petiolate anteriorly. Fore wing vein 2m-cu with an angulation (zig-zag) that is strong in most species (Figs 38–39) or weak (Fig. 7) and with one wide or two narrowly spaced bullae. Fore wing hyaline basally and infumate apically (Figs 38–39) or completely moderately infumate (Figs 7–8).

T1 gradually widening from anterior to posterior, 1.2 to 1.8 times as long as posterior width, with moderate anterolateral projections (as seen in dorsal view). Glymma present as a depression ventral to anterolateral projections. Dorsal longitudinal carinae of T1 present basally or extending up to 0.7 of segment. Dorsolateral longitudinal carinae of T1 present (most species) or absent; if present, not extending posterior to spiracle (Figs 40, 41) or extending up to 0.7 of segment, not bifurcated into two branches above and below spiracle. Spiracle of T1 just anterior to middle. T1 and T2 not fused. T2 without a postmedial transverse groove or grooves that delineate anterolateral corners. T2 to T4 completely yellow/orange, completely black, or black with yellow at posterior of each segment. Laterotergites wide (Fig. 42), of MS3 divided from tergite by a crease, of MS4 not divided. T7 and T8 not turned anteriorly under metasoma. Ovipositor straight, about equal to apical height of metasoma or a little shorter, carrying one egg (Figs 5, 45) or no eggs, dorsal valve tapered to a sharp point (Fig. 44). Ovipositor sheath in profile, widest basally, gradually tapering from base to apex, flexible from 0.3 to 0.6.

Body polished, face and supraclypeal area coarsely punctate (Figs 10–15); pronotum, mesopleuron and metapleuron with fine, sparse, but distinct punctures (Figs 30–33); all other body regions almost impunctate (Figs 34–37, 40–43). Body covered in dense setae, particularly long on supraclypeal area, shorter on pleura of mesosoma and metasomal tergites.

**Mature larva.** Unknown.

**Egg.** (Fig. 45): Stalk apical, anchor unknown, surface reticulate. Egg known only for *Zambion monodon* (holotype).

#### Hosts.

Unknown, but probably parasitoids of tenthredinoid sawflies (Hymenoptera) (see Introduction).

#### Distribution.

Afrotropical (Republic of Congo, Uganda, Kenya, Zambia and Angola).

#### Species included.

*Zambion monodon*, *Zambion hirtum* Delobel and five new species described below.

#### Comments.

*Zambion*, *Ibornia* and *Thibetoides* are all closely related based on seven synapomorphies, especially the thickened dorsoposterior corner of the pronotum and the supra-antennal prominence (Bennett 2002). Kasparyan (1993) speculated that *Zambion* and *Ibornia* must be sister genera because of the unidentate mandibles and because they are both Afrotropical, but Bennett (2002) found that *Ibornia* and *Thibetoides* are sister taxa based on seven characters including the fusion of T1 and T2 and the ventral curving of T7 and T8 under the metasoma which makes *Zambion* the sister of these two genera.

#### Key to the world species of *Zambion*

**Table:** 

1	Longitudinal lamella present between toruli (Fig. 23). Mesosoma yellow to orange (Fig. 4) AND head with dark markings dorsally (Fig. 16)	*Zambion hirtum* Delobel
–	Lamella absent between toruli. Mesosoma with dark markings (Figs 5-9), OR if mesosoma completely yellow to orange (Fig. 46), then head also yellow to orange, without dark markings dorsally near ocelli and horn (Fig. 47)	2
2(1)	Propodeum lacking a ventral transverse carina extending from metasomal foramen to pleural carina (Figs 36, 48)	3
–	Propodeum with a ventral transverse carina extending from metasomal foramen to pleural carina (Figs 35, 37)	4
3(2)	Mesopleuron with dorsal half black and ventral half yellow (Fig. 5). Notaulus weak, but present	*Zambion monodon* Kasparyan
–	Mesopleuron completely yellow (Fig. 46). Notaulus absent	*Zambion kasparyani* sp. n.
4(2)	Propodeum with medial longitudinal carinae absent or incomplete anterior to posterior transverse carina. Supra-antennal area with a long, sub-parallel-sided/ weakly tapering horn in dorsal view (Figs 20, 21), length 1.2 to 1.3 times width of pedicel at midheight in lateral view (Fig. 27)	5
–	Propodeum with medial longitudinal carinae strong and complete anterior to posterior transverse carina (Fig. 34). Supra-antennal area with a short triangular horn in dorsal view (Figs 18, 19) length 0.3 to 0.5 times width of pedicel at midheight in lateral view (Fig. 26)	6
5(4)	Propodeum black (Fig. 8). Clypeus black, except brown at extreme apical edge (Fig. 14). Lateral abscissa of posterior transverse carina of propodeum roughly straight, meeting pleural carina at right angle at about midpoint between propodeal spiracle and posterior end of pleural carina (similar to Fig. 36)	*Zambion wahli* sp. n.
–	Propodeum orange except brown basolaterally (Fig. 9). Clypeus with apical half orange and basal half black (Fig. 15). Lateral abscissa of posterior transverse carina of propodeum strongly curving posteriorly where it meets pleural carina at an acute angle near posterior 0.2 of pleural carina (Fig. 37)	*Zambion broadi* sp. n.
6(4)	Legs completely dark brown (Fig. 7). Tergites 3 to 7 completely brown (Fig. 7). Malar space 0.7 times basal width of mandible. Carina extending from pleural carina of propodeum to propodeal spiracle complete	*Zambion eileenae* sp. n.
–	Fore and mid legs orange, hind coxa, trochanter and femur orange, hind tibia and tarsus dark brown, except yellow at base of hind tibia (Fig. 6). Tergites 3 to 7 with medioposterior ivory bands (Fig. 6). Malar space 0.4 to 0.5 times basal width of mandible. Carina extending from pleural carina of propodeum to propodeal spiracle absent	*Zambion rogeri* sp. n.

### Zambion monodon

Kasparyan

http://species-id.net/wiki/Zambion_monodon

**Figures 1–3.**
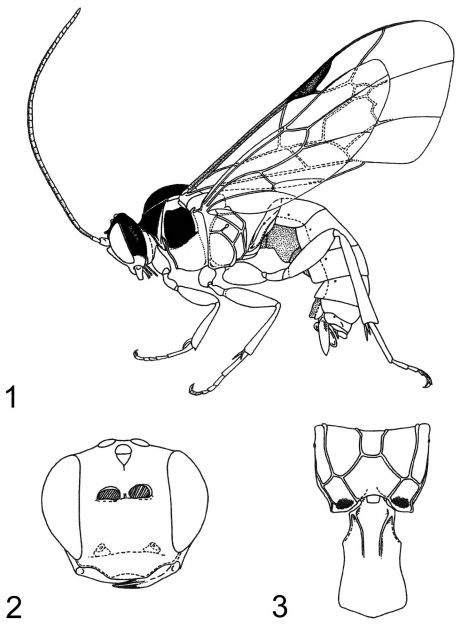
*Zambion monodon*,holotype female **1** habitus **2** head, anterior view **3** propodeum and first metasomal segment, dorsal view. Figure reproduced from Kasparyan (1993).

Figs 1–3 5 11 17 25 36 41, 42, 45

Zambion monodon  Kasparyan, 1993: 86.

#### Diagnosis.

*Zambion monodon* can be distinguished from all other *Zambion* spp. by having the mesopleuron approximately half black (dorsally) and half yellow (ventrally) (Fig. 5) (all other species are concolourous on mesopleuron – either all brown to black or all yellow to orange). *Zambion monodon* is unusual in *Zambion* in that it lacks the ventral transverse carina of the propodeum extending from the metasomal foramen to the pleural carina (Fig. 36). The only other species of *Zambion* that lacks this carina is *Zambion kasparyani*, but the latter species has a mesopleuron that is completely yellow to orange (Fig. 46).

#### Description.

**Adult**. Female (based on holotype only – see comments below). Fore wing length 7.2 mm. Clypeus separated from supraclypeal area by a strong groove (Fig. 11). Malar space 0.5 times basal width of mandible. Supraclypeal area 2.0 times as wide as high (Fig. 11), dorsomedially without a short, narrow, longitudinal lamella between the antenna. Supra-antennal horn moderately long and triangular in dorsal view (Fig. 17), about equal in length to width of pedicel at midheight in lateral view (Fig. 25). Ocello-ocular distance 1.4 times ocellar diameter. Gena 0.6 times transverse diameter of the eye. Antennal flagellum with 37 segments.

Notaulus present, but weak. Epicnemial carina medially curving slightly away from anterior edge of mesopleuron near ventral corner of pronotum (intermediate between *Zambion rogeri* shown in Fig. 32 and *Zambion wahli* shown in Fig. 33). Scutellum with lateral carinae 0.4 length of scutellum. Medial longitudinal carinae of propodeum complete and strong anterior to posterior transverse carina (Fig. 3). Carina extending from pleural carina to spiracle complete (Fig. 36). Posterior transverse carina of propodeum with lateral abscissa roughly straight, joining pleural carina at about 0.4 distance from posterior end of pleural carina to propodeal spiracle (Fig. 36). Ventral transverse carina extending from metasomal foramen to pleural carina absent (Fig. 36). Fore tibia with a strong apical point on dorsal surface. Hind femur 3.4 times as long as medial width (Fig. 1). Hind tarsal claw pectinate to about 0.5. Angulation of fore wing vein 2m-cu strong (Fig. 1).

Tergite 1 of metasoma 1.7 times as long as apical width. Dorsal longitudinal carinae of T1 extending posterior to spiracle (0.6 length of tergite), dorsolateral longitudinal carinae extending about half way to spiracle (0.3 distance of T1) (Fig. 41 of male, similar to female).

Yellow. Legs and metasoma become slightly darker yellow apically/ posteriorly. Scape, pedicel except apically, supra-antennal area except orbits ventral to medial ocellus, vertex, gena dorsal to ventral edge of eye, occiput, apex of mandibles, pronotum dorsal to furrow, mesoscutum, dorsal half of mesopleuron except subtegular ridge and scutellum except basolaterally black/ dark brown. Apex of pedicel, flagellum and distal tarsomere of hind leg brown. Entire membrane of fore wing moderately infumate (Fig. 5). Body covered with dense golden setae.

Male (based only on single male paratype noted by Kasparyan 1993): As female except fore wing length 7.0 mm. Malar space 0.4 times basal width of mandible. Width of supraclypeal area 1.7 times medial height. Ocello-ocular distance 1.2 times ocellar diameter. Gena 0.7 times transverse diameter of eye. Lateral abscissa of posterior transverse carina of propodeum joining pleural carina at 0.3 distance from posterior end of pleural carina. (Both antennae missing or incomplete).

**Figures 4–5.**
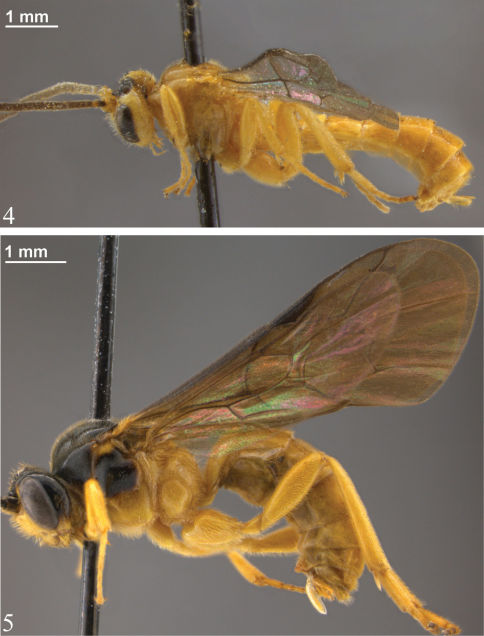
Habitus **4** *Zambion hirtum*,holotype female **5** *Zambion monodon*,holotype female.

#### Material examined.

Holotype: ♀, Label 1: ZAMBIA, Kitwe, Chati, 27.3.1979, K. Löyttyniemi leg. Label 2: window trap with *Eucalyptus*. Label 3: Holotypus *Zambion monodon* Kasparyan. Label 4: coll. Dept. Agr. Forest. Zool. Univ. Helsinki. [MZH]. Condition: intact except missing distal tarsomere of right foreleg and left mid leg detached and glued to top locality label. Paratypes: ♂, same data as holotype, except 8.iii.1979; ♀ same data except 15.iii.1979. (♀ paratype strongly damaged by Dermestidae).

#### Comments.

*Zambion monodon* appears related to *Zambion kasparyani* and *Zambion hirtum* on the basis of the similar moderately long, triangular-shaped supra-antennal horn (Figs 16, 17, 47). Within this grouping, *Zambion monodon* and *Zambion kasparyani* both lack the ventral transverse carina of the propodeum (Figs 36, 48). Outgroup comparison with *Ibornia* and *Thibetoides* spp., reveals that this character varies between species within these two genera as well, therefore its polarity (and phylogenetic utility) in *Zambion* is unclear. *Zambion monodon* is the only species of *Zambion* for which the egg is known. Kasparyan (1993) stated that the dermestid damaged paratype was a female. Because the metasoma posterior to T3 is missing, it is difficult to confirm the sex; however, the length of T1 (1.8 times posterior width) and ocellar-ocular distance (1.2 times ocellar diameter) suggest that this specimen may be a male based on comparison with the dimensions of these structures in the female holotype and male paratype.

### Zambion kasparyani sp. n.

urn:lsid:zoobank.org:act:C1C0F597-7D1C-4591-9E8C-4705AAE8F0AB

http://species-id.net/wiki/Zambion_kasparyani

Figs 46–48 

#### Diagnosis.

*Zambion kasparyani* can be distinguished from all other *Zambion* spp. by combination of both of the following characters: 1) absence of a ventral transverse carina on the propodeum extending from the metasomal foramen to the pleural carina (Fig. 48) (contrast with condition in *Zambion hirtum* shown in Fig. 35); 2) mesosoma completely yellow to orange (Fig. 46) (no dark colour).

#### Description.

**Adult.** Female unknown.

Male: Fore wing length 5.8 mm. Clypeus separated from supraclypeal area by a strong groove. Malar space 0.4 times basal width of mandible. Supraclypeal area 1.7 times as wide as high, dorsomedially without a short, narrow, longitudinal lamella between the antenna. Supra-antennal horn moderately long and triangular in dorsal view (Fig. 47), about equal in length to width of pedicel at midheight in lateral view. Ocello-ocular distance 1.2 times ocellar diameter. Gena 0.8 times transverse diameter of the eye. Antennal flagellum with 32 segments.

Notaulus absent. Epicnemial carina mostly straight medially, not curving away from anterior edge of mesopleuron near ventral corner of pronotum (similar to *Zambion rogeri* shown in Fig. 32). Scutellum with lateral carinae extending 0.4 length of scutellum. Medial longitudinal carinae of propodeum complete and strong anterior to posterior transverse carina (as in Fig. 3). Carina extending from pleural carina to spiracle complete (Fig. 48). Posterior transverse carina of propodeum with lateral abscissa roughly straight, joining pleural carina at about 0.4 distance from posterior end of pleural carina to propodeal spiracle (Fig. 48). Ventral transverse carina extending from metasomal foramen to pleural carina absent (Fig. 48). Fore tibia with a moderately strong apical point on dorsal surface. Hind femur 3.6 times as long as medial width. Hind tarsal claw pectinate to about 0.5. Angulation of fore wing vein 2m-cu strong.

Tergite 1 of metasoma 1.6 times as long as apical width. Dorsal longitudinal carinae of T1 extending to spiracle (0.4 length of tergite), dorsolateral longitudinal carinae extending just over half way to spiracle (0.3 distance of T1).

Yellow. Legs and metasoma slightly darker yellow apically/ posteriorly. Vertex and supra-antennal area medially, scape and mesoscutum orange. Pedicel and apex of mandible dark brown. Flagellum medium brown except base of first flagellomere orange. Entire membrane of fore wing moderately infumate (Fig. 46). Body covered with dense golden setae.

#### Material examined.

Holotype ♂, Label 1: KENYA: Rift Valley Province. Marich Pass Field Studies Centre, 1°32.2'N, 35°27.4'E, 13–14 June 2000. Label 2: M.H. Bourbin, V.H. Lee & W.J. Pulawski collectrs [CAS]. Condition: intact.

#### Etymology.

This species is named in honour of Dr. Dmitriy Kasparyan (Zoological Institute, Russian Academy of Sciences, St. Petersburg) for his contributions to ichneumonid taxonomy especially being the first to recognize and describe the genus *Zambion*.

#### Comments.

see comments for *Zambion monodon* and *Zambion hirtum*.

### Zambion hirtum

Delobel

http://species-id.net/wiki/Zambion_hirtum

Figs 4 10 16 22–24 30 34–35, 38 40, 43–44

Zambion hirtum  Delobel, 1993: 267.

#### Diagnosis.

*Zambion hirtum* can be distinguished from all other *Zambion* spp. in that the supraclypeal area dorsomedially has a short, longitudinal lamella between the antennae (Fig. 23) (lamella absent in all other species). In addition, *Zambion hirtum* is the only species that has the following colour combination: mesosoma and metasoma completely light coloured (yellow or orange) (Fig. 4) AND head yellow with black markings dorsally (Fig. 16).

#### Description.

**Adult.** Female (only holotype known). Fore wing length 7.1 mm. Clypeus separated from supraclypeal area by a weak groove (Fig. 10). Malar space 0.5 times basal width of mandible. Supraclypeal area 1.7 times as wide as high (Fig. 10), dorsomedially with a short, narrow, longitudinal lamella between the antenna (Fig. 23). Supra-antennal horn triangular in dorsal view (Fig. 16), 1.0 times as long as width of pedicel at midheight in lateral view (Fig. 24). Ocello-ocular distance 1.6 times ocellar diameter. Gena 0.7 times transverse diameter of the eye. Antennal flagellum with 40 segments.

Notaulus absent. Epicnemial carina mostly straight medially (similar to *Zambion rogeri* shown in Fig. 32), not curving away from anterior edge of mesopleuron near ventral corner of pronotum. Scutellum with lateral carinae at base only. Medial longitudinal carinae of propodeum strong and complete anterior to posterior transverse carina (Fig. 34). Carina extending from pleural carina to spiracle absent. Posterior transverse carina of propodeum with lateral abscissa roughly straight (Fig. 34), point of union with pleural carina at about 0.2 distance from posterior end of pleural carina to propodeal spiracle. Ventral transverse carina extending from metasomal foramen to pleural carina present (Fig. 35). Fore tibia with a weak apical point on dorsal surface. Hind femur 3.2 times as long as medial width (original description = 3.8). Hind tarsal claw pectinate to 0.6 times length of claw. Angulation of fore wing vein 2m-cu strong (Fig. 38).

Tergite 1 of metasoma 1.6 times as long as apical width. Dorsal longitudinal carinae of T1 extending close to spiracles (about 0.4 times length of tergite), dorsolateral longitudinal carina extending just over half way to spiracles (similar to Fig. 40 of male *Zambion hirtum*).

Yellow. Pedicel, medial 0.5 of supra-antennal area except dorsomedial part of horn (Fig. 16) and medial 0.3 of occiput black to dark brown. Flagellum and dorsomedial area of supra-antennal horn brown. Medial 0.5 of vertex posterior to ocelli, mesoscutum and apical 2 tarsomeres of fore and mid legs and apical 3 tarsomeres of hind leg brownish yellow. Membrane of fore wing hyaline, except slightly infumate in apical 0.3 (Fig. 38). Body covered with dense golden setae.

Male: as female except fore wing length 4.9 to 7.1 mm. Malar space 0.4 times basal width of mandible. Width of supraclypeal area 1.7 to 2.0 times height. Ocello-ocular distance 1.7 to 1.9 times ocellar diameter. Gena 0.6 times transverse diameter of eye. Flagellum with 36 to 40 segments. Notaulus weak anteriorly in some specimens. Lateral abscissa of posterior transverse carina of propodeum slightly sinuous (Fig. 35) or completely straight (Fig. 34), joining pleural carina at 0.2 to 0.4 distance from posterior end of pleural carina. Hind femur length 3.0 to 3.4 times medial width. Hind tarsal claw pectinate in basal 0.4 to 0.5 of claw. Tergite 1 1.3 to 1.7 times as long as apical width. Dorsolateral longitudinal carina of T1 extending less than half way to spiracle (Fig. 40) or a little bit more than half. Colour as female except, in some specimens medial 0.5 of vertex light brown to dark brown and occiput more extensively brown (up to medial 0.5). Supra-antennal horn completely black to dark brown in some specimens.

#### Material examined.

Holotype: ♀, Label 1: CONGO, Brazzaville, Centre ORSTOM, 8 novembre 1986, A. DELOBEL coll. Label 2: Holotype. Label 3: *Zambion hirtum* A. DELOBEL det 1993. Label 4: Muséum Paris EY6006. [MNHN]. Condition: intact except left antenna missing distal 12 flagellomeres and right mid leg missing distal tarsomere. Paratypes: 3 ♂, same data as holotype except: 18.i.1987 [EY 6008]; 17–24.v.1987 [EY6009] and 21.xii.1986 [EY6011] [MNHN]; ♂ same data as holotype except: 17–24.v.1987 [EY 6012] [CNC]. Non-type material: ♂, ANGOLA: Quirimbo. v.1934. K. Jordan. B.M. 1934–1935 [NHM].

#### Comments.

*Zambion hirtum* is a mostly pale-coloured species, most similar in colour to *Zambion kasparyani*, although the latter species is completely yellow to orange dorsomedially on the head (Fig. 47), whereas *Zambion hirtum* is extensively black in this area (Fig. 16). *Zambion hirtum* is the species with the most known specimens (eight) and is the only species known from two countries. Note that only six of the eight specimens were examined in this study. Also note that the supraclypeal longitudinal lamella of *Zambion hirtum* is not homologous with the supra-antennal lamella of *Ibornia*. The latter is positioned much more dorsally on the head (extending nearly to the medial ocellus) and its dorsal margin is strongly thickened with a longitudinal groove (Townes, 1969).

### Zambion rogeri sp. n.

urn:lsid:zoobank.org:act:B4429CF0-E964-4A22-A273-944742403D9A

http://species-id.net/wiki/Zambion_rogeri

Figs 6 12 18 32 

#### Diagnosis.

**Figures 6–7.**
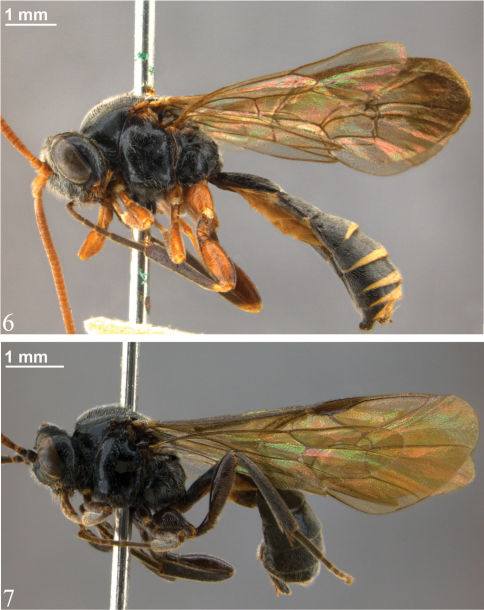
Habitus **6** *Zambion rogeri*,holotype male **7** *Zambion eileenae*,holotype female.

*Zambion rogeri* can be distinguished from all other *Zambion* spp. by having tergites 3 to 7 of metasoma black with ivory bands in posterior 0.1 to 0.2 (Fig. 6). No other species have banded metasomal segments.

#### Description.

**Adult.** Female: Unknown.

Male: Fore wing length 6.4 to 7.0 (6.4) mm. Clypeus separated from supraclypeal area by a strong groove (Fig. 12). Malar space 0.4 to 0.5 (0.5) times basal width of mandible. Supraclypeal area 1.7 to 1.9 (1.7) times as wide as high (Fig. 12), dorsomedially without a short, narrow, longitudinal lamella between the antenna. Supra-antennal horn short and broadly triangular in dorsal view (Fig. 18), 0.3 to 0.5 (0.3) times as long as width of pedicel at midheight in lateral view. Ocello-ocular distance 1.8 to 2.3 (1.8) times ocellar diameter. Gena 0.6 to 0.7 (0.6) times transverse diameter of the eye. Flagellum with 35 to 37 (35) segments.

Notaulus absent. Epicnemial carina mostly straight medially, not curving away from anterior edge of mesopleuron near ventral corner of pronotum (Fig. 32). Scutellum with lateral carinae 0.4 to 0.5 (0.5) length of scutellum. Medial longitudinal carinae of propodeum complete and strong anterior to posterior transverse carina (as in *Zambion hirtum* shown in Fig. 34). Carina extending from pleural carina to propodeal spiracle absent. Posterior transverse carina of propodeum with lateral abscissa roughly straight, point of union with pleural carina at about 0.4 distance from posterior end of pleural carina to propodeal spiracle (similar to *Zambion monodon* shown in Fig. 36). Ventral transverse carina extending from metasomal foramen to pleural carina present (as in Fig. 35). Fore tibia with a weak apical point on dorsal surface. Hind femur 3.5 to 3.7 (3.6) times as long as medial width. Hind tarsal claw pectinate to 0.4 times length of claw. Angulation of fore wing vein 2m-cu moderate.

Tergite 1 of metasoma 1.6 to 1.8 (1.6) times as long as apical width. Dorsal longitudinal carinae of T1 extending to about the level of spiracle or a bit beyond, 0.4 to 0.6 (0.6) times length of T1, dorsolateral longitudinal carinae extending to spiracle, or in holotype to at least 0.7 times length of T1 (posterior to this, present as a rounded ridge).

Black to dark brown. Extreme apex of hind femur, basal 0.2 of hind tibia and posterior 0.2+ of T3–T7 ivory (the ivory bands longer medially than laterally and increasing in length from T3 to T7) (Fig. 6). Scape, pedicel and flagellum ventrally, mandibles except at apex, palpi, pronotal collar medially, tegula, posterior of subtegular ridge, fore leg, mid leg, hind coxa, trochanter and femur orange (middle and hind trochanters and femora brownish orange, especially medial surface of hind trochanter and femur). Flagellum dorsally, apical 0.8 of hind tibia and all of hind tarsus brown (flagellum darkening from base to apex). Membrane of fore wing slightly infumate except moderately infumate in apical 0.3 (Fig. 6). Body covered with dense silver setae.

**Figures 8–9.**
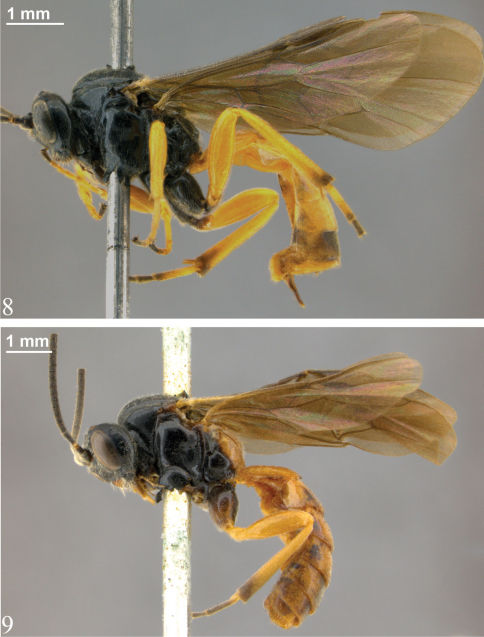
Habitus **8** *Zambion wahli*,holotype female **9** *Zambion broadi*,holotype male.

**Figures 10–15.**
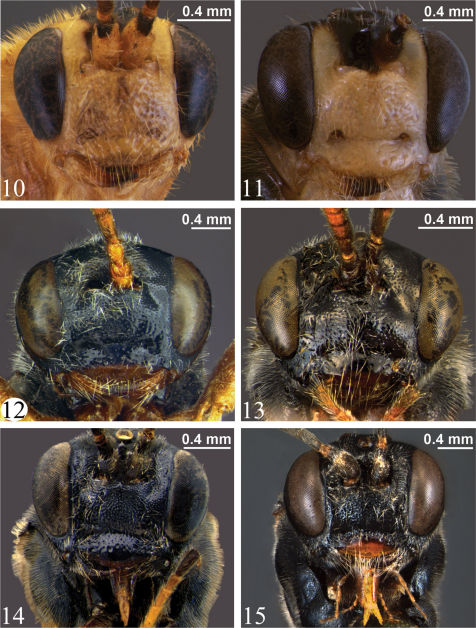
Head, anterior view **10** *Zambion hirtum*,paratype male **11** *Zambion monodon*, paratype male **12** *Zambion rogeri*,paratype male **13** *Zambion eileenae*,holotype female **14** *Zambion wahli*,paratype male **15** *Zambion broadi*, holotype male.

**Figures 16–21.**
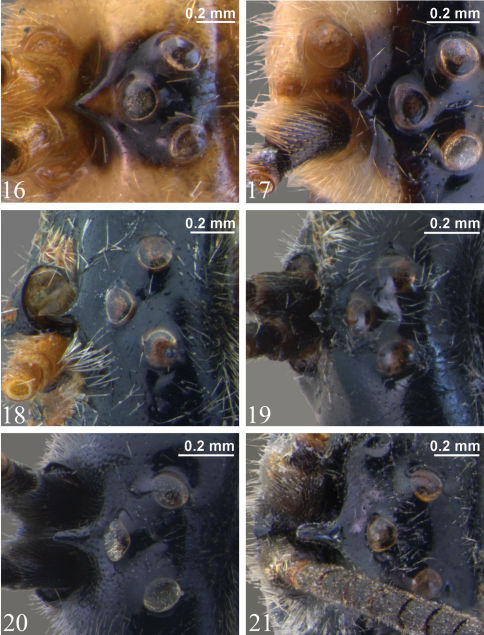
Vertex showing supra-antennal horn, dorsal view **16** *Zambion hirtum*, paratype male **17** *Zambion monodon*, paratype male **18** *Zambion rogeri*, paratype male **19** *Zambion eileenae*, holotypefemale **20** *Zambion wahli*, holotypefemale **21** *Zambion broadi*, holotype male.

#### Material examined.

Holotype: ♂, Label 1: BRIT, E, AFRICA [KENYA], Masai Reserve, 29.i.1914, Cap. A.O.Luckman. Label 2: 1919-10. [NHM]. Condition: intact except left antennal flagellum missing all but basal 24 flagellomeres. Paratypes: ♂, BRIT. E. AFRICA [KENYA], 30 miles from Magadi Junc., iv.1912. F.G. Hamilton [NHM]; ♂, BRIT. E. AFRICA [KENYA], Mogorr River, v.1913 [CNC].

#### Etymology.

This species is named in honour of the senior author’s father, Dr. Roger Bennett, for his support and encouragement during the long (and sometimes dark) journey towards becoming a systematic entomologist.

#### Comments.

*Zambion rogeri* is most closely related to *Zambion eileenae* on the basis of the similar short triangular horn of the supra-antennal area and the length of the longitudinal carinae of T1. Even though *Zambion rogeri* is only known from males, and *Zambion eileenae* from a single female, it is highly unlikely that they are conspecific. *Zambion rogeri* has extensive yellow colouration on the legs and ivory bands on the metasoma, whereas *Zambion eileenae* is completely dark. In the three species of *Zambion* for which both males and females are known, there are minimal sex-related colour differences and this is also the case for species in the related genera *Ibornia* (Seyrig 1935) and *Thibetoides* (Kasparyan 1973). In addition, the malar space of *Zambion rogeri* is 0.4 to 0.5 times the basal width of the mandible compared to 0.7 for *Zambion eileenae*. *Zambion rogeri* also lacks the carina extending from the pleural carina to the propodeal spiracle, whereas this carina is complete in *Zambion eileenae*.

### Zambion eileenae

sp. n.

urn:lsid:zoobank.org:act:E90E33CB-A2B1-4B1C-8A80-E0FCC2CD187

http://species-id.net/wiki/Zambion_eileenae

Figs 7 13 19 26 

#### Diagnosis.

*Zambion eileenae* can be distinguished from all other *Zambion* spp. by having all legs completely dark (brown or black), without any orange or yellow (Fig. 7). All other species have some orange or yellow in some parts of the legs.

#### Description.

**Adult.** Female. Fore wing length 6.0 mm. Clypeus separated from supraclypeal area by a strong groove (Fig. 13). Malar space 0.7 times basal width of mandible. Supraclypeal area 1.9 times as high as wide (Fig. 13), dorsomedially without a short, narrow, longitudinal lamella between the antenna. Supra-antennal horn short and triangular in dorsal view (Fig. 19), 0.3 times as long as width of pedicel at midheight in lateral view (Fig. 26). Ocello-ocular distance 1.8 times ocellar diameter. Gena 0.8 times transverse diameter of the eye. Flagellum with 29 segments on left, 28 on right.

Notaulus absent. Epicnemial carina mostly straight medially, not curving away from anterior edge of mesopleuron near ventral corner of pronotum (similar to *Zambion rogeri* shown in Fig. 32). Scutellum with lateral carinae extending 0.5 length of scutellum. Medial longitudinal carinae of propodeum complete and strong anterior to posterior transverse carina (as in *Zambion hirtum* shown in Fig. 34). Carina running from pleural carina to propodeal spiracle complete. Posterior transverse carina of propodeum with lateral abscissa roughly straight, point of union with pleural carina at about 0.4 distance from posterior end of pleural carina to propodeal spiracle (similar to *Zambion monodon* shown in Fig. 36). Ventral transverse carina extending from metasomal foramen to pleural carina present (as in Fig. 35). Fore tibia without an apical point on dorsal surface. Hind femur 3.6 times as long as medial width. Hind tarsal claw pectinate to about 0.5 times length of claw. Angulation of fore wing vein 2m-cu weak.

Tergite 1 of metasoma 1.8 times as long as apical width. Dorsal longitudinal carinae of T1 extending to 0.7 length of T1, dorsolateral longitudinal carinae extending beyond spiracles up to 0.7 length of segment.

Black. Legs, metasomal sternites and ovipositor sheaths at base brown, the legs lightening from base to apex except hind leg with tibia and tarsus darker brown. Flagellomere 1 at extreme base and flagellomeres 2+ on ventral surface yellow brown, gradually lightening towards apex, dorsal surface of flagellum brown, lightening to yellow brown apical to middle. Apical 0.3 of clypeus orange-brown. Membrane of fore wing strongly infumate (Fig. 7), slightly less imfumate basally. Body covered with dense silver setae.

Male: unknown.

**Figures 22–27.**
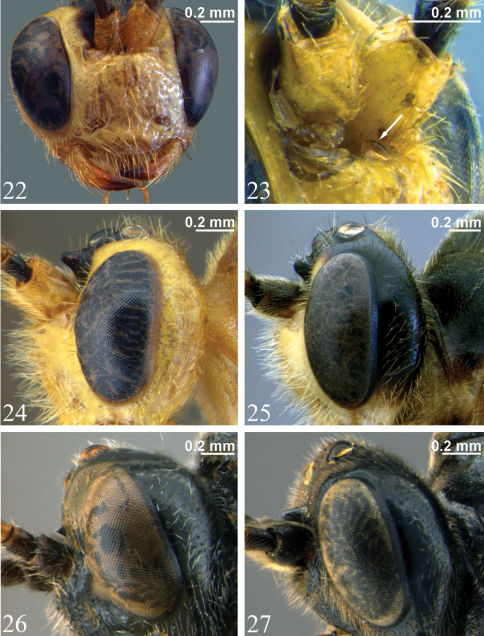
**22** Head, anterior view, *Zambion hirtum*, paratype male showing unidentate mandible **23** Supraclypeal area, anterolateral view, *Zambion hirtum*, male (non-type) (arrow points to longitudinal lamella between antennae) **24–27** head, lateral view **24** *Zambion hirtum*, paratype male **25** *Zambion monodon*, holotype female **26** *Zambion eileenae*,holotype female **27** *Zambion wahli*, holotypefemale.

#### Material examined.

Holotype: ♀, Label 1: UGANDA, Ruwenzori Range [Rwenzori Mountains], xii.1934-i.1935., B.M. E. Afr. Exp. B.M. 1935-203. Label 2: Namwamba Valley, 6,500 ft., F.W. Edwards. [NHM]. Condition: intact.

#### Etymology.

This species, collected in the “Mountains of the Moon”, is named in honour of the senior author’s mother, Mrs. Eileen Bennett, for her support and understanding, especially when finding dead caterpillars and grasshoppers in jars or worse (live ones wandering through the house).

#### Comments.

See comments for *Zambion rogeri*.

### Zambion wahli sp. n.

urn:lsid:zoobank.org:act:14B25D7C-F198-4BEE-B1C4-93FC0CAD4D41

http://species-id.net/wiki/Zambion_wahli

Figs 8 14 20, 27 28, 31, 33 

#### Diagnosis.

*Zambion wahli* can be distinguished from all other *Zambion* spp. by combination of all of the following characters: 1) mesopleuron completely black (no yellow) (Fig. 8); 2) supra-antennal horn long and sub-parallel-sided in dorsal view (Fig. 20), 1.2 to 1.3 times as long as pedicel at midheight in lateral view (Fig. 27); 3) propodeum completely black (Fig. 8) (not orange).

#### Description.

**Adult.** Female. Fore wing length 6.1 mm. Clypeus separated from supraclypeal area by a strong groove (Fig. 14). Malar space 0.5 times basal width of mandible. Supraclypeal area 2.1 times as wide as high (Fig. 14), dorsomedially without a short, narrow, longitudinal lamella between the antenna. Supra-antennal horn long and sub-parallel-sided in dorsal view (Fig. 20), 1.2 times as long as width of pedicel at midheight in lateral view. Ocello-ocular distance 1.6 times ocellar diameter. Gena 0.8 times transverse diameter of the eye. Antenna incomplete (see Material examined).

Notaulus present, but weak. Epicnemial carina medially curving away from anterior edge of mesopleuron near ventral corner of pronotum (Fig. 33) (not mostly straight medially as in Fig. 32). Scutellum with lateral carinae to 0.3 length of scutellum. Medial longitudinal carinae of propodeum incomplete anterior to posterior transverse carina (present between posterior transverse carina and the level of the propodeal spiracles, but absent anterior to this point). Carina running from pleural carina to spiracle absent. Posterior transverse carina of propodeum with lateral abscissa roughly straight, point of union with pleural carina at about 0.4 distance from posterior end of pleural carina to propodeal spiracle (similar to *Zambion monodon* shown in Fig. 36). Ventral transverse carina extending from metasomal foramen to pleural carina present (as in *Zambion hirtum* shown in Fig. 35). Fore tibia with a moderately strong apical point on dorsal surface. Hind femur 3.8 times as long as medial width. Hind tarsal claw with pectination not known (both distal tarsomeres missing). Angulation of fore wing vein 2m-cu strong.

Tergite 1 of metasoma 1.2 times as long as apical width. Dorsal longitudinal carina of T1 extending to about level of spiracle (0.4 length of segment), dorsolateral longitudinal carina absent (no obvious carina visible between anterior part of dorsal longitudinal carina and spiracle).

Black. Distal trochantelli of fore and middle legs, hind trochanter except at base, all femora and tibiae except apical 0.1 of hind tibia, fore tarsomeres 1 - 4, middle tarsomeres 1 – 3, basal 0.8 of hind basal tarsomere, T1 – 4, T5 sublaterally, T6 except anteriorly and all sternites yellow. Palpi, glossa, basal trochantelli of fore and middle legs, base of hind basal trochantellus, apical 0.1 of hind tibia, distal tarsomere of fore and middle leg, all of hind tarsus except basal 0.8 of basal tarsomere, T5 medially and laterally, T6 anteriorly and ovipositor sheaths brown. Membrane of fore wing moderately uniformly infumate (Fig. 8). Body covered with dense, golden setae.

Male as female except: Fore wing length 6.2 to 6.3 mm. Malar space 0.4 to 0.5 times basal width of mandible. Supraclypeal area 1.9 to 2.0 times as wide as high. Supra-antennal horn 1.3 times width of pedicel at middle. Ocello-ocular distance 1.8 to 2.0 times ocellar diameter. Gena 0.8 to 0.9 times transverse diameter of eye. Flagellum with 27 segments. Hind femur length 3.7 to 3.8 times medial width. Hind tarsal claw pectinate to 0.5 to 0.6 length of claw. Posterior transverse carina of propodeum incomplete in middle. Tergite 1 1.3 to 1.4 times as long as apical width. Dorsal longitudinal carina extending to just anterior to spiracles or to level of spiracles (0.3 to 0.4 length of T1). Dorsolateral longitudinal carina of T1 absent or present as a thin, weak ridge that extends half way to spiracle. Colour as female except apical 0.2 of hind tibia, apical 0.5 to 0.9 of hind basal tarsomere brown. Tergites 1 to 4 yellow brown with irregular brown mottling, especially medially and anteriorly on each segment. Tergite 5 yellow laterally. Gonoforceps basally yellow, apically brown.

**Figures 28–33.**
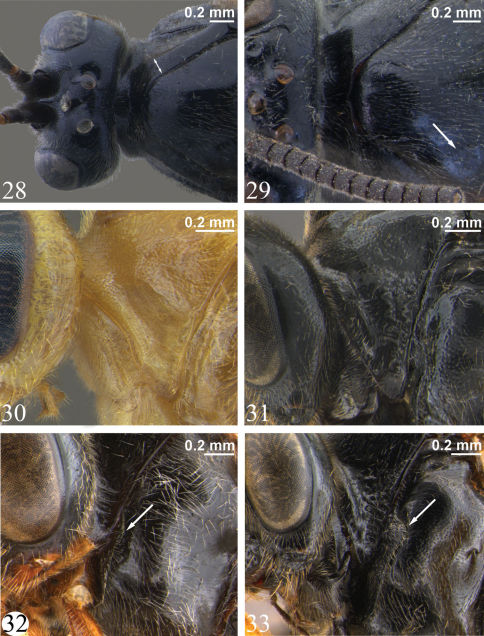
**28–29** Head, pronotum and anterior of mesoscutum, dorsal view **28** *Zambion wahli*, holotype female (double arrow shows widening of pronotum) **29** *Zambion broadi*, holotype male (arrow shows weak notaulus) **30–31** Pronotum, lateral view **30** *Zambion hirtum*, paratype male **31** *Zambion wahli*, holotype female **32–33**Mesopleuron, lateral view (arrow points to dorsal part of epicnemial carina) **32** *Zambion rogeri*, holotype male **33** *Zambion wahli*, holotype female.

#### Material examined.

Holotype: ♀, Label 1: A43. Label 2: AFRICA, UGANDA, Mt. Elgon, Butandiga, 7,000 ft. Label 3: J. Ford, B.M.1935-459. [NHM]. Condition: intact except both antenna with only basal flagellomere attached, right flagellomeres 2 to 14 glued to double-mount block, left hind leg missing distal 3 tarsomeres, right hind leg missing distal tarsomere, apical half of right ovipositor sheath missing. Paratypes: 2 ♂, same data as holotype except: A47 on upper label [NHM, CNC].

#### Etymology.

This species is named in honour of Dr. David Wahl (American Entomological Institute) (AEIC) for his contributions to ichneumonid taxonomy and his hospitality to the senior author during many visits to the AEIC.

#### Comments.

*Zambion wahli* is most closely related to *Zambion broadi* on the basis of the long, sub-parallel-sided horn (Figs 20, 21), the curved epicnemial carina (Fig. 33) and the lack of a complete medial, longitudinal carina of the propodeum anterior to the posterior transverse carina. See comments under the latter species for distinction of these two species.

### Zambion broadi sp. n.

urn:lsid:zoobank.org:act:920ECA3B-CC89-4A2E-B959-DD852CD33D7E

http://species-id.net/wiki/Zambion_broadi

Figs 9 15 21 29 37 

#### Diagnosis.

*Zambion broadi* can be distinguished from all other *Zambion* spp. by combination of all of the following characters: 1) mesopleuron completely black (without yellow) (Fig. 9); 2) supra-antennal horn long and sub-parallel-sided in dorsal view (Fig. 21), 1.2 times as long as pedicel at midheight in lateral view; 3) propodeum orange (not black) (Fig. 9).

#### Description.

**Adult.** Male. Fore wing length 6.4 mm. Clypeus separated from supraclypeal area by a strong groove. Malar space 0.4 times basal width of mandible. Supraclypeal area 1.9 times as wide as high (Fig. 15), dorsomedially without a short, narrow, longitudinal lamella between the antenna. Supra-antennal horn long and sub-parallel-sided in dorsal view (Fig. 21), 1.2 times as long as width of pedicel at midheight in lateral view. Ocello-ocular distance 2.1 times ocellar diameter. Gena 0.6 times transverse diameter of the eye. Flagellum with 30 segments.

Notaulus present, but weak (Fig. 29). Epicnemial carina medially curving away from anterior edge of mesopleuron near ventral corner of pronotum (similar to *Zambion wahli* shown in Fig. 33). Scutellum with lateral carinae at base only. Medial longitudinal carinae of propodeum absent anterior to posterior transverse carina. Posterior transverse carina incomplete medially. Carina extending from pleural carina to propodeal spiracle absent. Posterior transverse carina of propodeum with lateral abscissa strongly curving posteriorly where it joins pleural carina, point of union at about 0.2 distance from posterior end of pleural carina to propodeal spiracle (Fig. 37). Ventral transverse carina extending from metasomal foramen to pleural carina present (Fig. 37). Fore tibia apical point not examined (both fore tibiae absent). Hind femur 3.6 times as long as medial width. Hind tarsal claw pectinate to 0.4 times length of claw. Angulation of fore wing vein 2m-cu strong.

Tergite 1 of metasoma 1.2 times as long as apical width. Dorsal longitudinal carinae of T1 extending to about level of spiracle (0.4 length of tergite), dorsolateral longitudinal carinae present only as a short stub bifurcating near base of dorsal longitudinal carina.

Black. Apical half of clypeus, glossa, apex of scutellum, propodeum, metasoma, except as noted below, orange. Tegula, coxae, apical 0.2 of hind tibia, hind tarsus except basal 0.3 of basal tarsomere, T2+ in spots laterally and sublaterally, T5 to T6 medially and hypopygium brown. Palpi, hind trochanter, femur, basal 0.8 of hind tibia and basal 0.3 of basal tarsomere, sternites and gonoforceps brownish yellow. Note that the distinction between orange and brownish yellow is not always clear. The hind leg is slightly more yellowish than the propodeum, the latter being slightly more yellow (and slightly less orange) than the scutellum and tergites. Membrane of fore wing uniformly, moderately infumate (Fig. 9). Body covered with dense golden setae.

Female: unknown.

**Figures 34–39.**
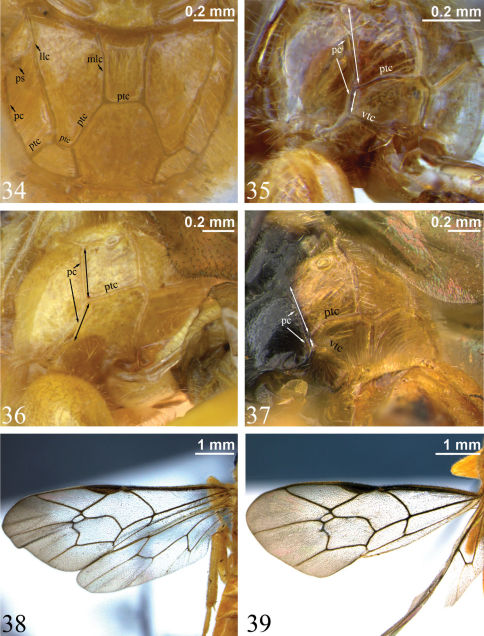
**34** Propodeum, dorsal view. *Zambion hirtum*, holotype female**35–37** Propodeum, dorsolateral view (double-headed arrows indicate length of pleural carina anterior and posterior to point of union with posterior transverse carina) **35** *Zambion hirtum*, paratype male **36** *Zambion monodon*, holotype female (note lack of ventral transverse carina) **37** *Zambion broadi*, holotype male **38–39** Fore wing **38** *Zambion hirtum*, holotype female **39** *Zambion hirtum*,paratype male. **llc** – lateral longitudinal carina, **mlc** – medial longitudinal carina, **pc** – pleural carina, **ptc** – posterior transverse carina, **ps** – propodeal spiracle, **vtc** – ventral transverse carina.

**Figures 40–45.**
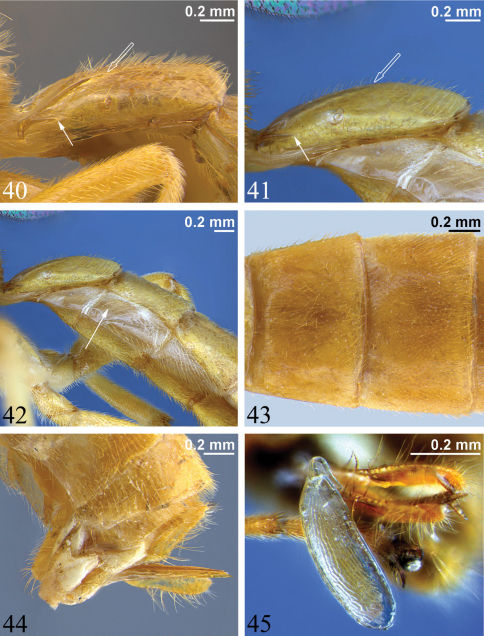
**40–41** First metasomal segment, lateral view (hollow arrow marks posterior end of dorsal longitudinal carina, solid arrow denotes posterior end of dorsolateral longitudinal carina) **40** *Zambion hirtum*, paratype male **41** *Zambion monodon*, paratype male **42** Anterior of metasoma, lateral view, *Zambion monodon*, paratype male (arrow points to laterotergite of second metasomal segment) **43** Tergites 2 and 3, dorsal view, *Zambion hirtum*, holotype female **44** Posterior of metasoma, lateral view showing ovipositor, *Zambion hirtum*, holotype female **45** Egg, lateral view, *Zambion monodon*, holotype female.

**Figures 46–48.**
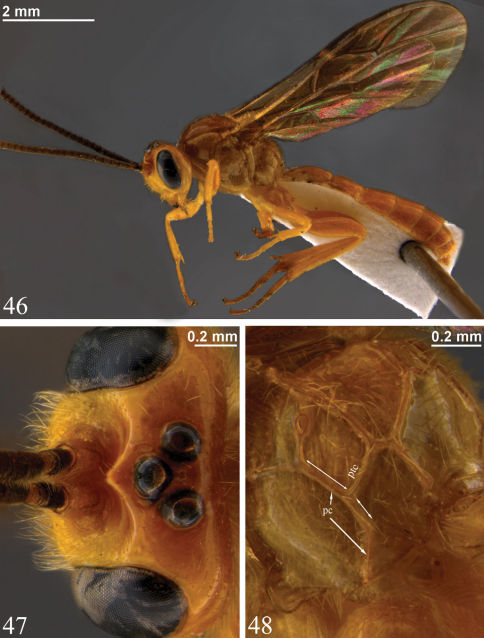
*Zambion kasparyani*, holotype male **46** Habitus **47** Head, dorsal view **48** Propodeum, dorsolateral view (note lack of ventral transverse carina similar to *Zambion monodon* in Fig. **36**).

#### Material examined.

Holotype: ♂, Label 1: BRIT. E. AFRICA [KENYA], 30 miles from Magadi Junc., April.1912, F.G. Hamilton. Label 2: 1915-98. [NHM]. Condition: right flagellum with only basal 15 flagellomeres, left flagellum with basal 5 flagellomeres attached, fore and middle legs missing except for coxae, left flagellomeres 6 to 15 and right hind leg glued on card below specimen.

#### Etymology.

This species is named in honour of Dr. Gavin Broad (NHM) in recognition of his contributions to ichneumonid taxonomy and for finding the type specimen of this species within the unidentified ichneumonids of the Natural History Museum, London.

#### Comments.

*Zambion broadi* is most closely related to *Zambion wahli* as noted under the comments for that species. The differences are the colour of the propodeum (compare Figs 8 and 9), the shape and location of the lateral abscissa of the posterior transverse carina of the propodeum, and the complete lack of the medial longitudinal carina dorsal to the posterior transverse carina of the propodeum in *Zambion broadi* (present, but incomplete in *Zambion wahli*). It is possible that *Zambion broadi* is conspecific with *Zambion wahli*, but we think this unlikely given the difference in both colour and propodeal carination. When both of these characters differ in related taxa, it is a good indicator that two species are present (e.g., in *Dyspetes* Förster) (He and Wan 1987).

## Supplementary Material

XML Treatment for Zambion

XML Treatment for Zambion monodon

XML Treatment for Zambion kasparyani

XML Treatment for Zambion hirtum

XML Treatment for Zambion rogeri

XML Treatment for Zambion eileenae

XML Treatment for Zambion wahli

XML Treatment for Zambion broadi
